# Evaluation of Different Registration Algorithms to Reduce Motion Artifacts in CT-Thermography (CTT)

**DOI:** 10.3390/diagnostics13122076

**Published:** 2023-06-15

**Authors:** Bogdan Kostyrko, Kerstin Rubarth, Christian Althoff, Miriam Zibell, Christina Ann Neizert, Franz Poch, Giovanni Federico Torsello, Bernhard Gebauer, Kai Lehmann, Stefan Markus Niehues, Jürgen Mews, Torsten Diekhoff, Julian Pohlan

**Affiliations:** 1 Department of Radiology, Charité—Universitätsmedizin Berlin, Humboldt-Universität zu Berlin, Freie Universität Berlin, 10117 Berlin, Germany; christian.althoff@helios-gesundheit.de (C.A.); giovanni.torsello@med.uni-goettingen.de (G.F.T.); bernhard.gebauer@charite.de (B.G.); stefan.niehues@charite.de (S.M.N.); torsten.diekhoff@charite.de (T.D.); julian.pohlan@charite.de (J.P.); 2 Institute for Biometry and Clinical Epidemiology, Charité—Universitätsmedizin Berlin, Humboldt-Universität zu Berlin, Freie Universität Berlin, 10117 Berlin, Germany; kerstin.rubarth@charite.de; 3 Berlin Institute of Health, Charité—Universitätsmedizin Berlin, Humboldt-Universität zu Berlin, Freie Universität Berlin, 10178 Berlin, Germany; 4 Department of General and Visceral Surgery, Charité—Universitätsmedizin Berlin, Humboldt-Universität zu Berlin, Freie Universität Berlin, 12203 Berlin, Germany; miriam.zibell@charite.de (M.Z.); christina.neizert@charite.de (C.A.N.); franz.poch@charite.de (F.P.); kai.lehmann@charite.de (K.L.); 5 Canon Medical Systems Europe BV, Global Research & Development Center, 2718 RP Zoetermeer, The Netherlands; juergen.mews@eu.medical.canon

**Keywords:** CT, computed tomography, thermoablation, CT thermography, CTT, registration, rigid, elastic

## Abstract

Computed tomography (CT)-based Thermography (CTT) is currently being investigated as a non-invasive temperature monitoring method during ablation procedures. Since multiple CT scans with defined time intervals were acquired during this procedure, interscan motion artifacts can occur between the images, so registration is required. The aim of this study was to investigate different registration algorithms and their combinations for minimizing inter-scan motion artifacts during thermal ablation. Four CTT datasets were acquired using microwave ablation (MWA) of normal liver tissue performed in an in vivo porcine model. During each ablation, spectral CT volume scans were sequentially acquired. Based on initial reconstructions, rigid or elastic registration, or a combination of these, were carried out and rated by 15 radiologists. Friedman’s test was used to compare rating results in reader assessments and revealed significant differences for the ablation probe movement rating only (*p* = 0.006; range, 5.3–6.6 points). Regarding this parameter, readers assessed rigid registration as inferior to other registrations. Quantitative analysis of ablation probe movement yielded a significantly decreased distance for combined registration as compared with unregistered data. In this study, registration was found to have the greatest influence on ablation probe movement, with connected registration being superior to only one registration process.

## 1. Introduction

Surgical resection is a common treatment for primary or secondary liver malignancies, but it is not an option for all patients [1,2]. On the one hand, surgery may be precluded due to patients’ comorbidities [2,3]. On the other hand, only 20% of metastatic liver tumors are resectable, which is why minimally invasive options such as thermal ablation are increasingly applied [1,3,4,5]. Various thermal ablation techniques are available, including microwave ablation (MWA) and radiofrequency ablation (RFA), which are widely used for malignant tumor ablation because of their high effectiveness and low complication rate [2,6]. Currently, it is recommended to use RFA for small tumors or as an alternative to surgery in cases of inappropriate tumor localization [7]. In the comparison of established RFA with MWA, no statistically significant differences in local recurrence could currently be shown [7]. The thermoablation technique is minimally invasive and can be performed percutaneously [2]. In this procedure, an ablation probe is inserted into the target area under image guidance, and applied heat causes necrosis of the tumor [2]. In most cases, the source of recurrence is in the marginal zone, where tumor tissue has not been completely ablated [8]. Therefore, it is important to monitor the success of the ablation in real time to ensure complete tumor absence.

To assess the success of the ablation, temperature measurement is required during the session to ensure that adequate tissue necrosis has been accomplished in the target area [9]. Noninvasive techniques for ablation zone monitoring include infrared thermal imaging (ITI), and laser Doppler, ultrasound (US)-, magnet resonance (MR)-, and computed tomography (CT)-based thermography (CTT) [9,10,11,12,13,14,15,16]. The ITI has the disadvantage that the tissue surface is not visible in the infrared camera to facilitate investigation of the temperature changes during ablation [13,14]. Therefore, only laparoscopic interventions can be considered as an option [13]. In comparison, laser Doppler measurement of blood flow within the ablated area allows conclusions to be drawn about necrosis [12]. However, blood flow within the ablation zone is irregular, and still present in the marginal zones, making it difficult to differentiate it from vital tissue and increasing the risk of recurrence [12]. For MR-guided procedures, metallic MWA and RFA probes are especially incompatible, so this thermography method is used for other ablation procedures [16]. As an alternative, hyperthermia can be monitored with ultrasound quickly, noninvasively, and without radiation exposure, but its sensitivity decreases above a temperature of 50 °C, making the evaluation of thermal ablation inaccurate in higher temperature ranges [15]. Since CT is already used for intervention planning and probe positioning, it could also be a good clinical option for temperature measurement during the procedure. CT-based temperature measurement exploits the well-established inverse relationship between Hounsfield units (HU) and temperature in different biological tissues [5,10]. Heating reduces the density of a medium, resulting in a decrease in HU [5]. This thermal sensitivity can predict whether the tissue in the ablation area has reached a temperature over 60 °C to induce coagulation necrosis or not [17]. Since several minutes can elapse between two CT scans, interscan motion artifacts induced by breathing, organ pulsation, or patient movement might degrade ablation zone monitoring. To minimize such artifacts, adequate registration of successively acquired images is necessary [5,6,18,19,20]

The aim of image registration is to match structures or features between the individual images to obtain diagnostic image quality [21,22]. Two major types of image registration algorithms are available: rigid and elastic registration [23]. In rigid registration, images are rotated and translated to achieve a match, which helps reduce artifacts due to object movement, such as the ablation probe [21]. Another advantage is that the images of the same object acquired at different times are averaged, thus increasing the signal-to-noise ratio [24]. On the other hand, elastic registration has more degrees of freedom and can additionally cope with deformations by localized stretching of images, allowing more precise matching of structures in several images [19,20,21,23]. This method best corrects breath-induced changes in the position of large organs such as the liver [19]. However, the correction becomes inaccurate when the patient takes a deep breath, so the artifacts cannot be corrected by the registration [19]. A breath-hold instruction for patients would be useful as an additional step in clinical practice [19]

The aim of this study was to evaluate different registration algorithms for CTT monitoring heat ablation of porcine liver in vivo.

## 2. Materials and Methods

### 2.1. Experimental Setup and Protocol

**Figure 1:**
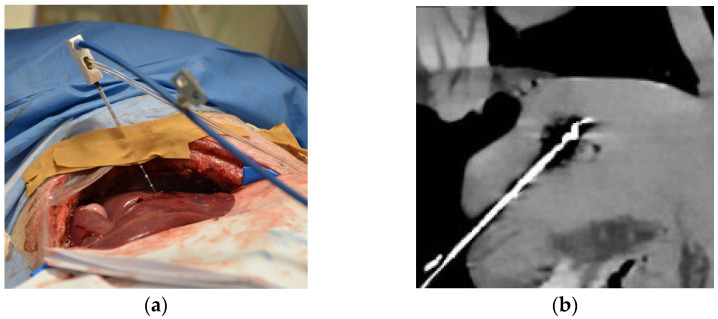
Experimental setup of computed tomography (CT)-based thermography (CCT): (**a**) Intraoperative photograph showing the animal under general anesthesia with the liver exposed and the microwave ablation (MWA) probe inserted; (**b**) CT image showing inserted MWA probe in the liver at the end of heating phase reaching maximum temperature (T_max_) before cooling down. In the same axis with a clear representation of the ablation probe and zone, the images for the rating were set.

Four MWAs were performed on normal liver tissue in a ventilated 6-month-old female domestic pig under general anesthesia. The animal was housed in the central animal husbandry of the Charité with compliance to 2010/63/EU-directives and recommendation of the GM-Solas (Society for Laboratory Animal Science, Freiburg, Germany) for pig husbandry. The experiments were conducted in 2020 using the latest CT technology as a proof of concept for further clinical trials. The porcine liver is similar to human liver parenchyma, and the artifacts due to respiration and heart action serve as a realistic model. The liver was exposed in a sterile environment to ensure better access to the organ with the ablation and temperature probes (Figure 1a). For each of the three ablations successfully performed, the MWA probe (AveCure, MedWaves Incorporated, San Diego, CA, USA) power was set to 100 W. One CTT dataset of the liver was acquired without thermoablation. The probe was placed in porcine liver for the respective ablation in such a way that there was sufficient distance to the other ablation areas. During each MWA, 20 spectral acquisitions were acquired without table movement using 16 cm detector coverage (Canon Aquilion ONE Prism; Canon Medical Systems, Otawara, Japan). Scan parameters: rapid kVp switching between 80 and 135 kVp, 1 s rotation time, 500 mA tube current. The first scan was acquired right at the beginning of the ablation, and each further scan at 30 s intervals throughout the 5 min of ablation. Maximum temperature was reached when the 10th scan was acquired (T_max_), and the probe cooled down during the remaining session (Figure 1b). Another 10 CT scans after the energy gift of the MWA were acquired every 60 s, covering the postablation phase. The animal was euthanized at the end of the experiment, and the liver was removed for histopathological assessment.

### 2.2. Image Reconstruction and Registration

For each ablation procedure, 20 spectral acquisitions with a 0.5 mm slice thickness were obtained. Mono-energetic calculations of soft tissue characteristics with 75 keV and 135 keV were generated by the CT system. Only 75 keV calculations were used for our analysis. All 20 mono-energetic image volumes were combined into one base series for further registration. In a first step, rigid and elastic registration was performed separately using the unregistered datasets. For rigid registration, we selected the probe tip at T_max_ in the center of the ablation zone, and all 20 images were registered with correspondence on this selected point. With elastic registration, deformations of the tissue caused by interscan motion could be corrected. In a second step, two combined registration datasets were created: the rigid-elastic registration first used a rigid registration, and the result was used as input for the elastic registration and the elastic–rigid registration starts using an elastic registration, and the result was used as input for the rigid registration. A total of five datasets were thus generated for our analysis: unregistered image volume data as the benchmark, rigid and elastic registration, and then two combinations of both. These registrations were performed separately for each of the four CTT datasets, each including 20 image volume scans over the time covering the upslope and downslope phases (5 registration methods × 4 CTT datasets × 20 image volume scans, *n* = 400 image volumes).

### 2.3. Image Rating

All images were anonymized for blinded assessment and displayed with settings, ensuring that the ablation probe and the ablation zone were clearly appreciated (Figure 1b). The order of pooled CT images from three thermoablation datasets using different registration methods was manually randomized in the rating. Fifteen radiologists at different experience levels (mean ± 1 SD, 8.5 ± 6 years; range, 3–23 years) participated in the session using an in-house-developed rating tool on high-resolution monitors (Eizo RadiForce RX250, Eizo Corporation, Hakusan, Japan) [6,18,20,25,26]. Seven of the readers were interventional radiologists and were familiar with thermal ablation treatment (Table S1). The other eight readers were diagnostic radiologists (Table S1). The number of participants was chosen to achieve sufficient validity of the rating. For evaluation of the registration methods, the readers rated five criteria on a 10-point scale (Table 1). For the assessment, the readers were able to access all 20 images of the ablation procedure in one stack to actively scroll through the imaging data. The criteria were as follows: overall registration quality, movement of the ablation zone, movement of the ablation probe, tissue distortion, and image artifacts. As there was one CTT dataset with no thermoablation, it was removed from the statistics of the rating because no ablation area could be assessed.

### 2.4. Quantitative Analysis

For the quantitative analysis, the CT volume series of each ablation were set parallel to the probe axis so that it was visible in its full length. Regions of interest (ROIs) were then placed at the tip of the probe at T_max_ (10th scan), when the ablation zone was large enough to identify the tip properly. The software measured the distance between the ROIs at T_1_ and T_2_, T_2_, T_3_, etc., between any two CT scans of the series, resulting in 19 ROI distance measurements (in mm) for each series of 20 CT scans. The same approach was also used for registered datasets. Since the ablation probe was placed in the tissue and the CT volumes were acquired according to protocol, quantitative measurement was performed, including all four CTT datasets [27].

### 2.5. Statistics

The planning of the entire statistical evaluation was carried out with biometric expert consulting. Friedman’s test was performed to test for statistically significant differences in subjective scores between five dependent samples, in this case, thermoablation datasets with different registration algorithms. Friedman’s two-way ANOVA was used for a multiple comparisons test, analyzing differences in means in each rating criterion to show whether the difference in the score of one registration method compared with other methods was significant. An intra-class correlation coefficient (ICC) was calculated for interrater reliability, and the Koo/Li scale was used for interpretation [28]. A one-way ANOVA and Levene’s test were performed to determine if there were overall differences in mean values and variances of ROI movement between the registration methods investigated, respectively. The above statistical analysis was performed using IBM SPSS software (version 27.0, Armonk, NY, USA). Bonferroni’s multiple comparison test was used to compare ROI movement between registered and unregistered datasets. Descriptive statistics were used to calculate mean values and standard deviations. The analyses described above were carried out using Graphpad Prism software (Version 9.3.1, San Diego, CA, USA). A *p* value < 0.05 was considered significant. 

## 3. Results

### 3.1. Subjective Assessment of Image Registration

**Figure 2:**
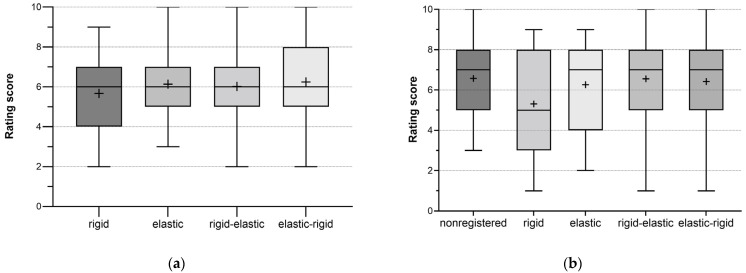
Boxplots of scores assigned for overall registration quality and ablation probe movement. Plus (+): mean value (**a**) Overall registration quality; *n* = 240; 3 ablations × 20 time points per ablation × 4 registrations; 15 readers. (**b**) Assessment of ablation probe movement; *n* = 300; 3 ablations × 20 time points per ablation × 5 (4 registrations + nonregistered dataset); 15 readers.

No statistically significant difference in overall registration quality was found between the individual registration algorithms investigated (Figure 2a). The readers rated all methods similarly, with an average of 6 points, so the quality of registration did not depend on a particular registration technique (Table 2). Although rigid registration showed a slightly worse score, this result was statistically non-significant, with *p* = 0.206. Other evaluation criteria were also tested for the significance of the differences between the rating scores of individual registration methods (Friedman’s test). A significant difference (*p* = 0.006; range, 5.3–6.6 points) was found for ablation probe movement only, meaning that assessed registration algorithms were rated significantly different for this criterion (Table 3). The participating expert readers concluded that the movement of the ablation probe was not compensated well enough with rigid registration rating this registration algorithm, with a lower score than the other methods (Figure 2b). The pairwise comparisons with all other registration algorithms were statistically significant (*p* < 0.05). In contrast, the differences between the individual registrations for all other criteria were statistically non-significant (*p* > 0.05).

The agreement on the overall performance as well as the majority of specific criteria (Table 4) was good. In the separate analysis of each criterion, good interrater reliability was also found for overall registration quality, ablation zone movement, and ablation probe movement. Image artifacts showed moderate agreement between readers. Only the tissue distortion criterion yielded poor agreement.

### 3.2. Quantitative Analysis

**Figure 3:**
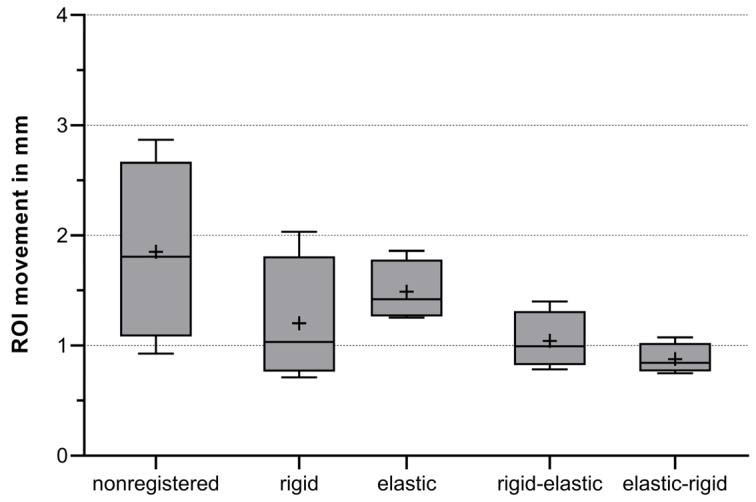
Boxplots of measured probe tip movement in mm. Mean value of region of interest (ROI) movement was measured for comparison, including all four CTT datasets acquired. Mean values (+) were calculated of all 19 ROI-distance measures for each ablation and registration algorithm, respectively. *n* = 400; 4 CTT datasets × 20 time points per ablation × 5 (4 registrations + nonregistered); *p* < 0.001 for differences between mean values and *p* < 0.001 for variances.

Quantitative analysis of probe movement was evaluated in all registration modalities to verify the results of the subjective analysis. It can be deduced that registered images are superior to unprocessed data (Figure 3). In the analysis, it was found that the movement of the ablation probe tip during CTT is more pronounced when only a single registration is applied (mean ± 1 SD, rigid = 1.2 mm ± 0.6 mm; elastic = 1.5 mm ± 0.3 mm). However, when a double registration is performed, this movement is better compensated and is less intense (mean ± 1 SD, rigid-elastic = 1.0 mm ± 0.3 mm; elastic-rigid = 0.9 mm ± 0.1 mm). Bonferroni’s multiple comparison test showed the difference in mean values between connected registration and nonregistered data to be highly significant, with *p* < 0.001.

## 4. Discussion

In this study, we compared two registration algorithms and a combination of them against unregistered data acquired with CTT monitoring during MWA performed in a pig. Qualitative analysis showed overall good interrater agreement and revealed a statistically significant difference in subjective assessment of ablation probe movement with rigid registration being marginally inferior to the other registration methods. Quantitative analysis showed that image registration helps to reduce ablation probe tip movement during CTT. However connected registration showed more movement reduction (mean ± 1 SD, rigid–elastic = 1.0 mm ± 0.3 mm; elastic–rigid = 0.9 mm ± 0.1 mm) than one process registration (mean ± 1 SD, rigid = 1.2 mm ± 0.6 mm; elastic = 1.5 mm ± 0.3 mm).

In contrast to this experimental setup, most likely fewer CT volumes will be acquired in the clinical CTT setting, as radiation dose reduction is an aim for physicians and patients [3,5,10]. For the evaluation of the ablation result in CTT, at least two scans are required: one before the start of the procedure and one at the end. Since the time interval between these two serial scans can be several minutes, depending on the duration of the ablation, registration algorithms are required to accurately evaluate the ablation zone. In addition, the MWA probe is located at the center of the ablation zone, and the ablated area is assessed by the probe axis, so it is important to compensate for the movement of the probe between CT scans. Our experimental setting shows that image registration, especially connected registration, is effective for significantly reducing motion artifacts and should be evaluated in clinical practice. In the late developmental phase of CCT, it should also be possible to perform the registration directly on the CT to save time during the intervention and to quickly evaluate the need for post-ablation.

Minimizing motion artifacts in CT scans acquired during thermal ablation procedures has both diagnostic and therapeutic relevance [22]. Different registration algorithms are available for motion correction and have been used to minimize changes induced by breathing, heartbeat, or other involuntary movements [18]. However, additional methods can also be used to reduce the change in patient position between image acquisitions. These include instructing the patient to hold their breath, if possible, with some fixation in a certain position, and optimizing the CTT protocol [19,22]. In several previous studies investigating and comparing different registration algorithms for motion correction, rigid registration showed the poorest results in both the subjective rating and quantitative analysis compared with other investigated registration methods [18,19,20,22,29,30]. While connected registration algorithms have also previously been investigated, no study has so far compared connected registration with single registrations or unregistered data [29].

The recruitment of 15 readers for the evaluation of CT images provides a sufficient basis to achieve relevant results. However, a larger number of readers could lead to even better and more significant results by considering a wider variety of opinions and perspectives. While interrater agreement was generally high, some criteria, such as tissue distortion and image artifacts, showed low interrater reliability. We assume that the criteria were insufficiently defined and are subject to individual interpretation. Only one CT scanner was used in our experiment. The results may therefore differ, and the transfer to other systems is difficult. We also applied and compared the most common registration algorithms. Thus, it is possible that further research will reveal better methods for thermoablation using different imaging modalities or software algorithms. The study is based on an animal experiment in which only one protocol was investigated. Furthermore, only one animal and three liver lesions were examined, which is also a limiting factor. Nevertheless, the model is suitable for applying the acquired knowledge about registration algorithms in clinical studies on humans. The radiation dose in clinical practice may be much lower than it was in our experiment. This could result in different image quality and higher image noise, which can make it difficult to assess the ablation zone or register the data. Furthermore, the evaluation of the registration methods was based on 20 CT scans per thermoablation, which is reduced in the clinical scenario to two scans. Therefore, the CT images used to assess the success of the ablation procedure are more likely to be affected by motion artifacts at a greater time interval between two scans. As has already been mentioned, additional tools, such as a breath-hold protocol, are likely to be required.

Unfortunately, the rating assessment did not reveal significant differences between the individual registration methods for all criteria. However, registration was found to have the greatest influence on probe movement. Our results show statistically significant differences between registered and unregistered CT data acquired during thermal ablation procedures, which is why registration is essential. Connected registration of both algorithms performed better than a one-time registration process in terms of subjective qualitative scores, which are also supported by quantitative analysis. Furthermore, other registration methods and their combinations can be investigated to further improve CTT. While our experiment provides useful data on the performance of different registration algorithms, further clinical trials are desirable to confirm our findings.

## 5. Summary

Thermal ablation is considered an alternative treatment option for patients with malignant tumors. To reduce the rate of recurrence, it is critical to evaluate the success of ablation. Different methods for non-invasive monitoring of thermoablation are investigated, considering their advantages and disadvantages. Because CT scans are already used for treatment planning, CT-based thermography can also be used. However, artifacts caused by motion, respiration, or organ pulsation may occur because several minutes may elapse between CT scans. Registration can be used to minimize these artifacts. There are two main methods with different degrees of freedom and application areas: rigid and elastic registration.

In this study, two main registration methods and their combinations were investigated. In vivo experiments with three microwave ablations on a healthy porcine liver were used as input, with 20 monoenergetic volumes acquired per ablation. Five data sets were assessed: an unregistered series, a single rigid or elastic registered series, and a double registered series with rigid–elastic and elastic–rigid registration. The images were assessed by 15 radiologists in a blinded rating setting using defined criteria. Quantitative analysis was then performed by placing an ROI at the tip of the probe and assessing probe movement during ablation.

Qualitative analysis showed that registration had the greatest impact on ablation probe movement for raters. The differences in the evaluation of other criteria were not significant after application of Friedman’s test, although inter-rater reliability was good. With quantitative measurement, it could be confirmed that, especially, double registration best compensates for the movement of the probe.

In this preclinical in vivo study, registration of 20 CT volumes per ablation was performed, which cannot be imagined in the clinical setting due to the high radiation exposure. During the intervention on patients, pre- and post-interventional scans were acquired, and registration of both scans is essential to minimize motion artifacts. Furthermore, additional methods, such as breath-hold protocols, can be applied to provide a better result. The rating assessment of this study could not reveal significant differences for each criterion. However, it was found that registration minimizes the movement of the ablation probe, which was also confirmed with quantitative analysis. The results provide essential information for the application of CTT that needs to be corroborated in a prospective clinical study.

## Figures and Tables

**Table 1:** Rating criteria were all specifically addressed during the reading. The table specifies the evaluation scoring system, ranging from 1 to 10 (in points).

Criterion	Lowest Score of 1	Highest Score of 10
Quality of the registration	poor registration quality	excellent registration quality
Ablation zone movement	zone moves around	zone is fixed in image
Ablation probe movement	probe moves around	probe fixed in image
Tissue distortion	distinct tissue distortions	no tissue distortions
Image artifacts	distinct image artifacts	no image artifacts

**Table 2:** Rating scores for each criterion by registration algorithm. Data presented as mean scores ± 1 SD for all three succeed ablations and all 15 readers on scale from 1 to 10 (in points).

Criterion/ Registration	Registration Quality	Zone Movement	Probe Movement	Tissue Distortion	Image Artifacts
non-registered	6.5 ± 1.9	6.9 ± 1.9	6.2 ± 1.9	6.8 ± 1.7	5.5 ± 1.8
rigid	5.6 ± 1.7	6.3 ± 1.9	5.3 ± 2.4	5.9 ± 2.2	4.8 ± 2.0
elastic	6.1 ± 1.7	6.7 ± 1.8	6.3 ± 2.1	6.8 ± 1.7	5.2 ± 1.7
rigid-elastic	6.0 ± 1.8	6.9 ± 1.7	6.6 ± 2.1	6.2 ± 1.9	4.9 ± 1.7
elastic-rigid	6.3 ± 1.8	6.8 ± 1.8	6.6 ± 2.2	6.4 ± 1.9	5.2 ± 1.8

**Table 3:** Differences in the assessment of each rating criterion according to different registration algorithms. Friedman’s test was performed to check all criteria for statistically significant differences in the rating of the registration modes; *p* values are provided.

Criterion	*p* Value	Minimal Rating Score (in Points)	Maximal Rating Score (in Points)
Quality of the registration	0.206	5.6	6.5
Ablation zone movement	0.239	6.3	6.9
Ablation probe movement	0.006	5.3	6.6
Tissue distortion	0.217	5.9	6.8
Image artifacts	0.077	4.8	5.5

**Table 4:** Reader agreement based on intra-class correlation coefficients. The scale of Koo/Li was used for interpretation [28]. Results are depicted as k-values with 95% confidence intervals (95% CI).

Criterion	k-Value	95% CI	Koo/Li-Scale
Overall	0.810	0.731–0.871	good
Quality of the registration	0.772	0.574–0.906	good
Ablation zone movement	0.774	0.580–0.907	good
Ablation probe movement	0.880	0.768–0.952	good
Tissue distortion	0.335	−0.047–0.691	poor
Image artifacts	0.524	0.194–0.792	moderate

## Data Availability

The study data are available upon request.

## Supplementary Materials

The following supporting information can be downloaded: https://www.mdpi.com/article/10.3390/diagnostics13122076/s1, Table S1: Reader details.
