# Supplementary material for: Evaluation of Different Registration Algorithms to Reduce Motion Artifacts in CT-Thermography (CTT)
Source: Diagnostics (Basel). 2023 Jun 15;13(12):2076. doi: 10.3390/diagnostics13122076 (PMC10296896; doi:10.3390/diagnostics13122076)
Supplement: Supplementary file 1 [file diagnostics-13-02076-s001.zip › diagnostics-2382635-supplementary.pdf]

Supplementary Table S1: Reader details.

Reader No.	Level of experience in years	Focus
Reader 1	11	intervention
Reader 2	8	intervention
Reader 3	5	intervention
Reader 4	6	diagnostic
Reader 5	5	diagnostic
Reader 6	4	diagnostic
Reader 7	3	diagnostic
Reader 8	5	diagnostic
Reader 9	23	intervention
Reader 10	21	intervention
Reader 11	6	intervention
Reader 12	6	diagnostic
Reader 13	7	diagnostic
Reader 14	6	diagnostic
Reader 15	12	intervention
